# A U-shaped protection of altitude against mortality and infection of COVID-19 in Peru: an ecological study

**DOI:** 10.1186/s12889-023-15537-7

**Published:** 2023-06-01

**Authors:** L. Baquerizo-Sedano, L. Goni, C. Sayón-Orea, P. González-Muniesa

**Affiliations:** 1 Facultad de Ciencias de la Salud, Universidad Privada del Norte, Lima, Peru; 2 Faculty of Pharmacy and Nutrition, Department of Food Sciences and Physiology, University of Navarra, Pamplona, Spain; 3 Center for Nutrition Research, University of Navarra, Pamplona, Spain; 4 Department of Preventive Medicine and Public Health, University of Navarra, Pamplona, Spain; 5 IDISNA – Navarra Institute for Health Research, Pamplona, Spain; 6 CIBER Physiopathology of Obesity and Nutrition (CIBERobn), Carlos III Health Institute (ISCIII), Madrid, Spain; 7 Navarra Public Health Institute, Pamplona, Spain

**Keywords:** COVID-19, Altitude, Risk factor, Hypoxia

## Abstract

**Background:**

The COVID-19 pandemic has affected the world in multiple ways and has been a challenge for the health systems of each country. From the beginning, risk factors for the severity and mortality of the disease were considered, as the spread of the virus was related to the living conditions of each population.

**Methods:**

In this ecological study we have evaluated the role of geography, precisely the altitude above sea level in the incidence and mortality of COVID-19 in Peru. Incidence and mortality data were taken from the open-access database of the government of Peru until March 2021. COVID-19 cases and COVID-19 mortality were treated as cases/density population and 1000 x cases/inhabitants while altitude was treated as continuous and as a categorical variable divided in 7 categories. The relationship between COVID-19 cases or deaths for COVID-19 and altitude as continuous variable was determined using Spearman correlation test. Meanwhile when altitude was considered as a categorical variable, Poisson regression or negative binomial analyses were applied.

**Results:**

A significant inverse correlation was found between COVID-19 cases by population density and altitude (r=-0.37 p < 0.001). By altitude categories, the lowest risk for infection was observed between 3,000 and 3,500 m (IRR 0.08; 95% CI 0.05,0.12). Moreover, we found an inverse correlation between altitude and COVID-19 mortality (r=-0.39 p < 0.001). Also, the lowest risk for mortality was observed between 3,000 and 3,500 m (IRR 0.12; 95%CI 0.08; 0.18). Similar results were found when analyses were adjusted for inhabitants and stratified by sex.

**Conclusion:**

This study reports an inverse relationship between COVID-19 incidence and mortality with respect to the altitude of residence, particularly, a u-shaped protection is shown, with a highest benefit between 3000 and 3500 m. The possibility of using hypoxia as an alternative treatment requires more complex studies that should allow knowing the physiological and environmental mechanisms of the protective role.

**Supplementary Information:**

The online version contains supplementary material available at 10.1186/s12889-023-15537-7.

## Introduction

The pandemic caused by SARS-CoV-2 started in December 2019 in China and spread to 195 countries affecting more than 670 million people and causing more than 6.8 million deaths [1]. The incidence and mortality among different countries varied due to a complex interaction between factors [2], some related to the particularity of each country such as geography, population density, altitude levels, humidity and temperature [3], others related to the population [4] such as migration [5, 6], socioeconomic status [7], health status and presence of comorbidities; as well as the public policies use [6, 8]. In addition to conditions related to the precision of the data such as the amount and type of diagnostic tests used, the report of cases to the coordinating institution and in relation with mortality, the capacity of health attention for each place. In most countries an underestimation of mortality has been observed [9]. Real data on mortality placed Peru as one of the countries with the highest number of deaths per million inhabitants [1].

Since the beginning of the pandemic, obesity is known to be the main modifiable factor in relation to the severity of infection and mortality from SARS-CoV-2 [10]. This would be due to the chronic low-grade inflammatory condition associated with the dysfunctional accumulation of adipose tissue [11]. Similarly, people with diseases such as diabetes, hypertension, cardiovascular diseases, and other chronic conditions were more susceptible to develop a severe clinical case due to the infection [12]. On the other hand, there is evidence on the beneficial effect of altitude hypobaric hypoxia against these metabolic related pathologies [11, 13–17].

Oxygen is essential for life and evolutionarily mechanisms have been developed to maintain cellular homeostasis of this molecule. The main regulator of oxygen delivery and utilization is the hypoxia-inducible factor 1 (HIF-1), and the activity of prolyl hydroxylases 1,2 and 3 (PHDs). The HIF-1 activity modulates the expression of hundreds of gene products in response to hypoxia or ischemia [18, 19]. In this sense, hypoxia inhibits the expression of ACE2, a receptor of SARS-CoV-2, that has been related to a lower incidence and severity of the infection [20–22]. For this reason, a possible protective role of hypoxia could be considered against the COVID-19 incidence and mortality. In any case, the link with high-altitude hypoxia is still under investigation.

Peru is a country with a very particular geography with populated areas at low altitudes (coast and jungle) but also a significant number of medium and large cities located at high altitudes > 1500 meters above sea level (m), which makes it ideal for studying natural conditions of hypobaric hypoxia. Several studies carried out from the beginning of the pandemic observed a protective role of altitude on COVID-19 infection [23, 24] and an improvement in survival amongst people admitted to the intensive care unit [25]. However, a small study (n = 159) carried out at La Rinconada, in southern Peru at 5300 m (the highest-altitude city in the world) fail to confirm this observation [26].

Therefore, the aim of this study was to analyze the COVID-19 incidence and mortality according to the altitude level of residence and to find optimal levels of altitude as a protective factor.

## Methods

### Study design

The present study is an ecological study based on data derived from open-access databases managed by the Ministry of Health from Peru. We collected the number of COVID-19 cases from the https://www.datosabiertos.gob.pe/dataset/casos-positivos-por-covid-19-ministerio-de-salud and deaths from https://www.datosabiertos.gob.pe/dataset/fallecidos-por-covid-19-ministerio-de-salud-minsa which was obtained until march 2021. These data were selected up to this timeframe because large-scale vaccination began in Peru in April 2021, which may have subsequently influenced findings.

For the data of positive cases, it is the daily record of positive cases of COVID-19 confirmed with any type of test and that present symptoms. Each record is equal to one person, which can be characterized by gender, age, and geographic location down to the district level [27].

For the mortality data, according to the Ministry of Health of Peru, each record is equal to a person, which can be characterized by sex, age, and geographic location up to the district level [28]. Since this dataset was published, each record represented a deceased confirmed by COVID-19, who met at least one of the following seven technical criteria:

*Virological criterion*: Death in a confirmed case of COVID-19 who dies within 60 days after a molecular (PCR) or reactive antigen test for SARS-CoV-2. *Serological criterion*: Death in a confirmed case of COVID-19 who dies within 60 days after a positive IgM or IgM/IgG serological test for SARS-CoV-2. *Radiological criterion:* Death in a probable case of COVID-19 that presents a radiological, tomographic or nuclear magnetic resonance image compatible with COVID-19 pneumonia. *Epidemiological link criterion*: Death in a probable case of COVID-19 that has an epidemiological link with a confirmed case of COVID-19. *Epidemiological investigation criteria*: Death in a suspected case of COVID-19 that is verified by epidemiological investigation of the National Epidemiology Network (RENACE). *Clinical criteria*: Death in a suspected case of COVID-19 with a clinical picture compatible with the disease. *SINADEF criteria*: Death with death certificate in which the diagnosis of COVID-19 is presented as the cause of death. Death due to COVID-19 on the death certificate is defined by the presence in fields A, B, C or D of the ICD-10 codes: U071, U072, B342, B972, or the mention of the terms “coronavirus”, “cov-2”, “cov2”, “covid” and “sars”.

### Measures

The COVID-19 cases and mortality database included a total of 196 provinces and the altitude ranged from 5 m (Talara, Zorritos) to 4342 m (Cerro de Pasco) of altitude (Supplementary Table 1).

The dependent variables were COVID-19 cases and COVID-19 mortality. Both measures were treated as cases/density population for the main analyses and 1000 x cases/inhabitants for the secondary analyses. The independent variable was altitude and was treated as continuous and as a categorical variable divided in 7 categories: <1500 m, 1500-1999 m, 2000-2499 m, 2500-2999 m, 3000-3499 m, 3500-3999 m, and ≥ 4000 m.

Population data, population density, and altitude of capital of provinces by provinces in Peru were collected from the *Centro Nacional de Planeamiento Estratégico (CEPLAN)* which was updated in December 2017 [29].

### Statistical analysis

The relationship between COVID-19 cases or deaths for COVID-19 and altitude, as continuous variable, was determined using Spearman correlation test. Meanwhile when altitude was considered as a categorical variable, Poisson regression or negative binomial regression analyses were applied according to the residual analysis of the Poisson regression, which was performed using the goodness of fit test and also plotting Pearson residuals and deviance residuals vs. the fitted values (Supplementary Figs. 1 and 2). The analyses by 1000 x cases/inhabitants were conducted by total population and stratified by sex. Statistical analyses were performed using STATA 16, and a P value < 0.05 was considered as statistically significant.

## Results

By March 2021, a total of 1,583,371 COVID-19 positive cases were recorded. For analysis, a total of 82,635 subjects without information of province were excluded. Therefore, the final number of cases was 1,500,736. Of them 51.1% were male and 48.9% female. The mean age of individuals was 42.3 (SD 17.8) years.

**Fig. 1:**
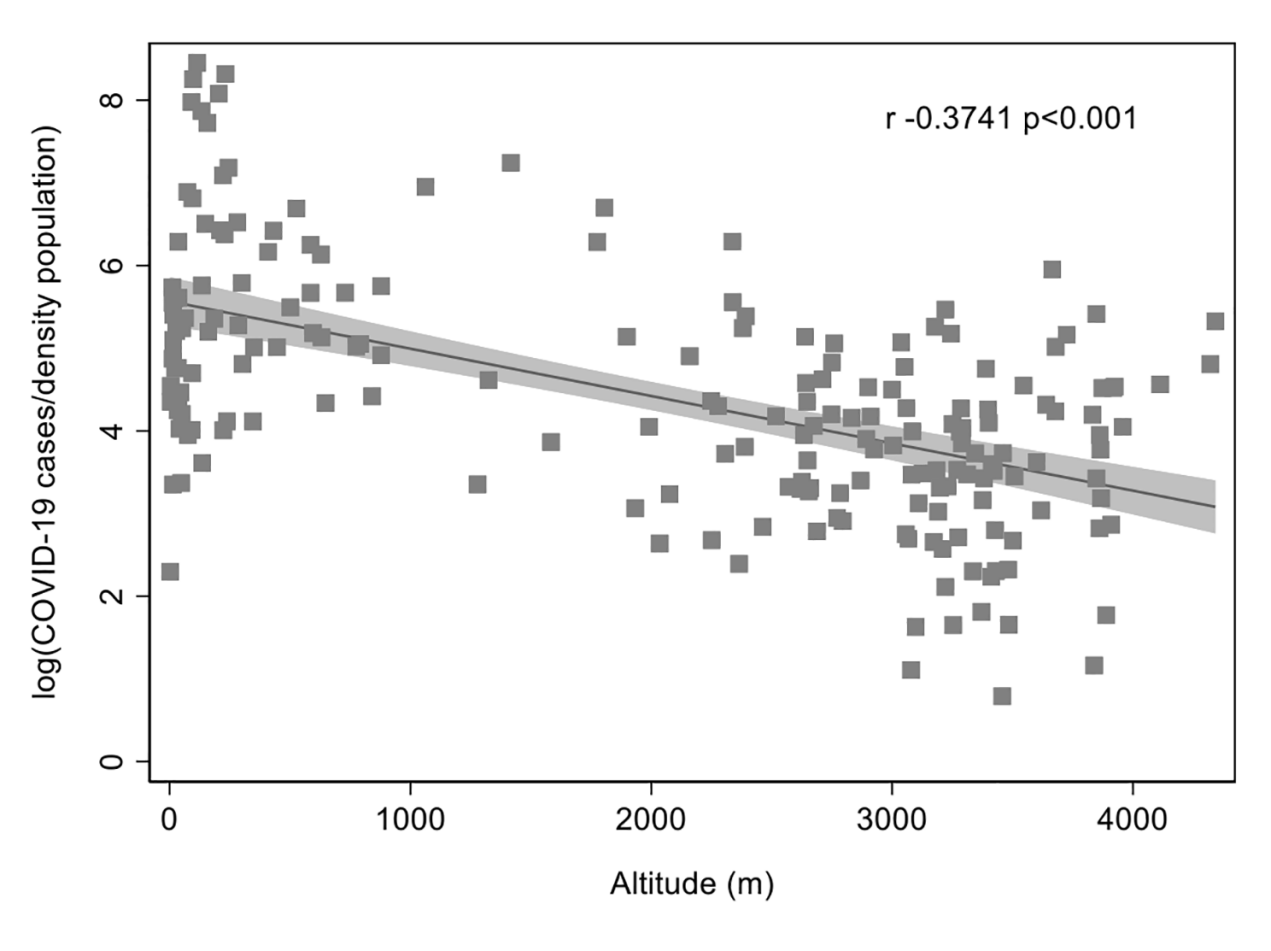
Association between COVID-19 cases by density population in Peru in relation to altitude (m)

We have found that the number of positive cases for COVID-19 depends on altitude of residence. A significant inverse correlation was found between COVID-19 cases by population density and altitude (r=-0.37 p < 0.001) **(**Fig. 1**)**. A stronger correlation was found by 1000 x COVID-19 cases/inhabitants (r=-0.53 p < 0.001) (Supplementary Fig. 3A). Once the population was stratified by sex, the same pattern was observed (Supplementary Fig. 3B and 3 C).

**Fig. 2:**
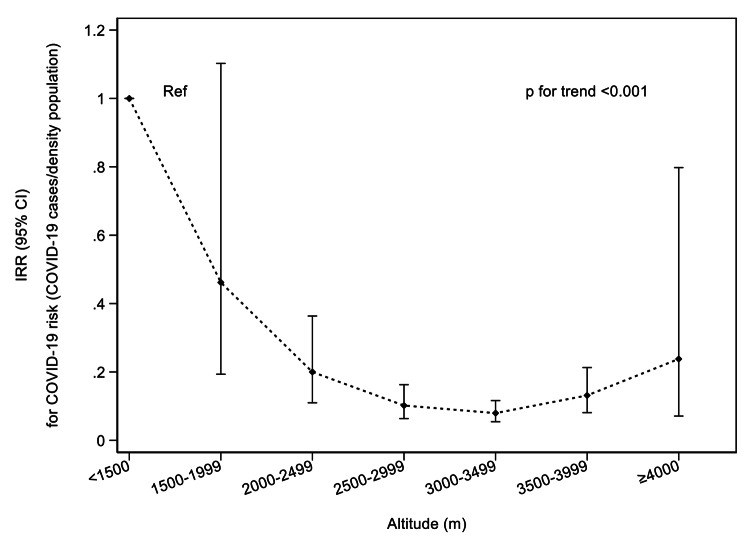
Negative binomial regression analyses of COVID-19 cases by density in Peru in relation to altitude (m). *IRR, Incidence-rate ratio; 95% CI, 95% Confidence Interval*

Figure 2 shows the risk of COVID-19 across 7 altitude categories (< 1500 m, from 1500 to 4000 m every 500 m, and ≥ 4000 m) obtained from negative binomial regression analysis. The highest altitude vs. lowest shows the risk of COVID-19 by density population (IRR_< 1500 m vs. ≥4000m_0.24; 95% CI 0.07,0.08; p trend < 0.001). In this sense, the biggest protective effect of altitude against COVID-19 was found in the 3000-3500 m category (IRR_< 1500 m vs. ≥3000−3500 m_ 0.08; 95% CI 0.05,0.12). Analyses by 1000 x COVID-19 cases/inhabitants showed similar results in the overall population and by sex (Supplemental Fig. 4). The analyses on the incidence were carried out with the frequently used altitude categories [30] showing the same tendency (Supplementary Table 2).

**Fig. 3:**
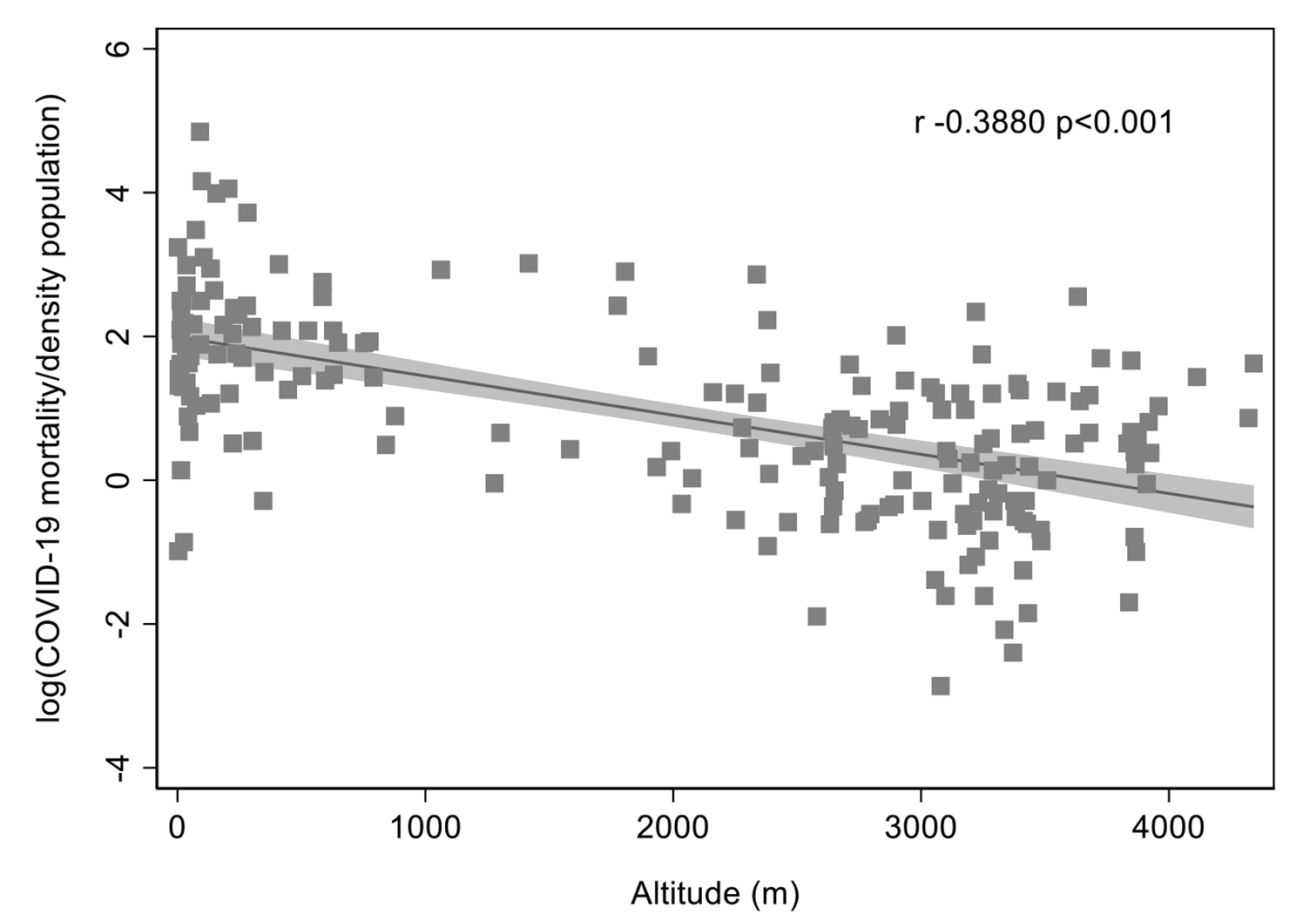
Association between COVID-19 mortality by density population in Peru in relation to altitude (m)

The total number of deaths across all provinces of Peru, by March 2021, was 50,831. A total of n = 238 subjects did not have information of province listed, therefore the final total number of deaths was 50,593. Similar to the results for COVID-19 cases, we found an inverse correlation between altitude and deaths by COVID-19 by density population (r=-0.39, p < 0.001) (Fig. 3). Additional analyses by 1000 x COVID-19 mortality/inhabitants and stratified by sex did not change the results (Supplementary Fig. 5).

**Fig. 4:**
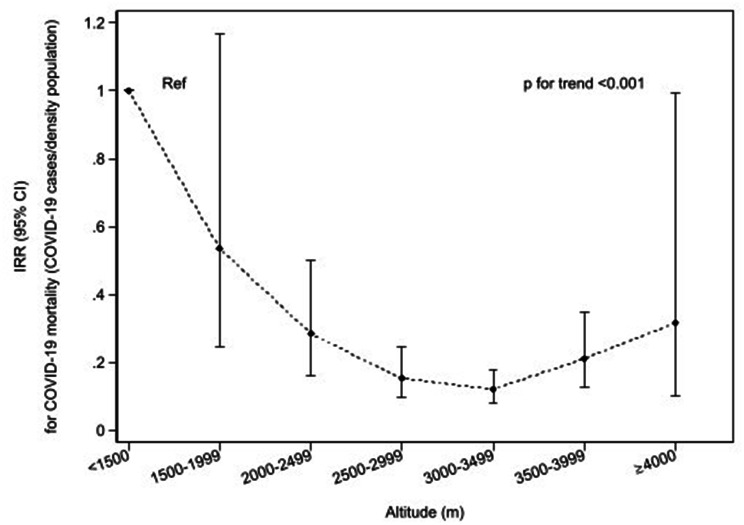
Negative regression analyses of COVID-19 mortality by density population in Peru in relation to altitude (m). *IRR, Incidence-rate ratio; 95% CI, 95% Confidence Interval*

According to the negative binomial regression analysis, the risk of death was lower at higher altitudes (IRR_< 1500 m vs. ≥4000 m_ 0.31; 95% CI 0.10,0.99; p trend < 0.001) (Fig. 4). The biggest protective effect of altitude against COVID-19 mortality risk was found in the 3000-3500 m category (IRR_< 1500 m vs. ≥3000−3500 m_ 0.12; 95%CI 0.08;0.18). Similar results were found when the mortality was adjusted by inhabitants and population was stratified by sex (Supplementary Fig. 6). The analyses with the frequently used altitude categories [30] were carried out, showing the same tendency on mortality (Supplementary Table 3).

## Discussion

In this ecological study, the most relevant finding was the protective role of altitude against the incidence and mortality from COVID-19, especially between 3000 and 3500 m. This finding is relevant because a significant number of people (500 million) live at more than 1500 m [31], and physiological adaptations to chronic exposure and its effect on mortality is constantly being studied [32]. In this sense, shortly after the start of the COVID-19 pandemic, the first reports were presented on the possible protective role of residence at high altitudes [33]. In addition, from the beginning of the pandemic, various strategies were used to reduce contagion, the main one was to reduce social contact; thus, it was necessary to include population density in the analyses due to its role in the spread of the virus [34] as well as the difference by sex. Despite the restrictions, initially there was a great migration from the capital to the interior of the country, which could be a factor in the rapid spread of the virus [24].

Our results are in accordance with other studies carried out worldwide [21, 23, 24, 35–39], also, this issue has been addressed on diverse populations with multicenter studies [40] and have even considered the geographic spread of the virus [24]. The opposite results to initial studies [40] may be attributed to the use of only three altitude categories (less than 1,500, 1,500-1,900 and more than 2,000 m) in addition to the lack of adjustment for population density.

This protection can be explained by physiological and anatomical adaptations to chronic exposure to lower partial pressure of oxygen, typical of high altitudes, as the better response to inflammation mediated by a better regulation of IL-6 and nitric oxide [41] better pulmonary perfusion and capacity [42] lower expression of ACE2 [20], and the increased of EPO [43]. Although previous studies have shown that patients with COVID-19 residing in high-altitude tend to have higher levels of inflammatory cytokines, particularly IL-6, IL-10 and TNF-α [44]. However, other conditions typical of high altitude, such as radiation and air humidity, must also be considered as variables of the protective role [37]. A study conducted in Ecuador did not find a lower viral load related to altitude perhaps because they did not consider the time of development of the disease or the peak of the viral load, the state of the immune system or the use of medication that can reduce the viral load [45].

Another important finding is that protection against COVID-19 begins to decrease from 3500 m. This could be due to the higher incidence of chronic mountain illness, which includes arterial hypoxemia, blood hyperviscosity and thrombosis, factors associated with higher morbidity and mortality [46, 47]. These pathological conditions also have been related with a poorer prognosis of COVID-19 [48, 49].

### Strength and limitations

In contrast to previous studies, we use the official open database, which reduced the underestimation of incidence and mortality, analyzing the 196 provinces in Peru, considering the altitude of the capital. Moreover, we censured the database until the start of the vaccination, March 2021. Also, there are limitations associated with our study. The prevalence of non-communicable diseases was not considered for correction or the age of the population. Another important limitation inherent to the ecological studies includes lack of access to smaller unit-sized data and individual data such BMI or abdominal perimeter, considering that chronic inflammation due to excessive accumulation of adipose tissue is one of the best identified risk factors for severity of disease, it is a possible confounding variable. However, the spread of the virus is also related to their own living conditions, socioeconomic status, compliance with the rules of confinement and social isolation, basic sanitary conditions and, of course, migration. Finally, the care capacity in hospitals or health centers at the provincial or departmental level was not considered either because the data is not available.

## Conclusion

An inverse relationship was found between COVID-19 incidence or mortality and the altitude of residence, when considering the latter as a continuous variable, this finding is supported by other similar studies. In our opinion, the most differential finding seems to be a u-shaped protection, showing that the altitude between 3000 and 3500 m is the optimal range against COVID-19 incidence and mortality. The possibility of using hypoxia as an alternative treatment requires more complex studies that allow knowing the physiological and environmental mechanisms of the protective role.

## Electronic supplementary material

Below is the link to the electronic supplementary material.

Supplementary Material 1

## Data Availability

All data about of number of COVID-19 cases and deaths in Peru is available in the official web of government of Peru: https://www.datosabiertos.gob.pe/dataset/casos-positivos-por-covid-19-ministerio-de-salud-minsa. Geographical population and altitude data for each city are available in the official web of government of Peru: https://www.ceplan.gob.pe/informacion-sobre-zonas-y-departamentos-del-peru/. The datasets used during the current study are available from the corresponding author on reasonable request. In any case the databases that we use are public.
