# Supplementary material for: A U-shaped protection of altitude against mortality and infection of COVID-19 in Peru: an ecological study
Source: BMC Public Health. 2023 Jun 1;23:1054. doi: 10.1186/s12889-023-15537-7 (PMC10234586; doi:10.1186/s12889-023-15537-7)
Supplement: Supplementary file 1 — Supplementary Material 1 [file 12889_2023_15537_MOESM1_ESM.docx]

**SUPPLEMENTARY MATERIAL**

**Supplementary table 1.** Population, population density and altitude by provinces in alphabetical order.

| **Provinces** | **Population** | **Population density (inhabitants/km^2^)** | **Altitude (meters above sea level)** |
| --- | --- | --- | --- |
| ABANCAY | 106753 | 31 | 2392 |
| ACOBAMBA | 80849 | 89 | 3431 |
| ACOMAYO | 27530 | 29 | 3221 |
| AIJA | 7719 | 11 | 3427 |
| ALTO AMAZONAS | 122343 | 6 | 148 |
| AMBO | 56712 | 36 | 2076 |
| ANDAHUAYLAS | 170725 | 43 | 2901 |
| ANGARAES | 64545 | 33 | 3271 |
| ANTA | 56571 | 30 | 3345 |
| ANTABAMBA | 13369 | 4 | 3640 |
| AREQUIPA | 991218 | 102 | 2337 |
| ASCOPE | 121266 | 46 | 238 |
| ASUNCION | 8707 | 16 | 3336 |
| ATALAYA | 54629 | 1 | 228 |
| AYABACA | 141469 | 27 | 2748 |
| AYMARAES | 33146 | 8 | 2911 |
| AZANGARO | 138382 | 28 | 3865 |
| BAGUA | 77053 | 14 | 421 |
| BARRANCA | 148801 | 109 | 51 |
| BELLAVISTA | 60821 | 8 | 262 |
| BOLIVAR | 16553 | 10 | 3098 |
| BOLOGNESI | 33181 | 11 | 3401 |
| BONGARA | 34334 | 12 | 1991 |
| CAJABAMBA | 80555 | 45 | 2651 |
| CAJAMARCA | 393120 | 132 | 2719 |
| CAJATAMBO | 7681 | 5 | 3382 |
| CALCA | 74797 | 17 | 2925 |
| CALLAO | 1042496 | 7145 | 3 |
| CAMANA | 60128 | 15 | 15 |
| CANAS | 39689 | 19 | 3910 |
| CANCHIS | 102643 | 26 | 3546 |
| CANDARAVE | 7995 | 4 | 3460 |
| CANETE | 239386 | 52 | 28 |
| CANGALLO | 33905 | 18 | 2570 |
| CANTA | 15447 | 9 | 2833 |
| CARABAYA | 98296 | 8 | 4321 |
| CARAVELI | 41972 | 3 | 1776 |
| CARHUAZ | 47554 | 59 | 2632 |
| CARLOS FERMIN FITZCARRALD | 21863 | 35 | 3079 |
| CASMA | 48236 | 21 | 45 |
| CASTILLA | 38459 | 6 | 631 |
| CASTROVIRREYNA | 19150 | 5 | 3958 |
| CAYLLOMA | 99582 | 7 | 3632 |
| CAÑETE | 239386 | 52 | 28 |
| CELENDIN | 96003 | 36 | 2645 |
| CHACHAPOYAS | 55476 | 17 | 2339 |
| CHANCHAMAYO | 209362 | 44 | 775 |
| CHEPEN | 89225 | 78 | 135 |
| CHICLAYO | 870930 | 265 | 34 |
| CHINCHA | 222338 | 74 | 94 |
| CHINCHEROS | 59246 | 48 | 2795 |
| CHOTA | 164502 | 43 | 2387 |
| CHUCUITO | 151552 | 38 | 3868 |
| CHUMBIVILCAS | 83036 | 15 | 3678 |
| CHUPACA | 53494 | 47 | 3281 |
| CHURCAMPA | 44628 | 37 | 3275 |
| CONCEPCION | 56015 | 18 | 3286 |
| CONDESUYOS | 17570 | 3 | 2935 |
| CONDORCANQUI | 55704 | 3 | 222 |
| CONTRALMIRANTE VILLAR | 20348 | 10 | 5 |
| CONTUMAZA | 31836 | 15 | 2647 |
| CORONEL PORTILLO | 386142 | 10 | 157 |
| CORONGO | 8102 | 8 | 3173 |
| COTABAMBAS | 53114 | 20 | 3292 |
| CUSCO | 458570 | 744 | 3414 |
| CUTERVO | 140312 | 46 | 2628 |
| DANIEL ALCIDES CARRION | 54889 | 29 | 3199 |
| DATEM DEL MARANON | 65792 | 1 | 133 |
| DOS DE MAYO | 54119 | 37 | 3210 |
| EL COLLAO | 86288 | 15 | 3862 |
| EL DORADO | 41639 | 32 | 346 |
| ESPINAR | 69763 | 13 | 3924 |
| FERREÑAFE | 107699 | 68 | 42 |
| GENERAL SANCHEZ CERRO | 29039 | 5 | 2160 |
| GRAN CHIMU | 31268 | 24 | 1279 |
| GRAU | 26438 | 12 | 3376 |
| HUACAYBAMBA | 23104 | 13 | 3191 |
| HUALGAYOC | 103134 | 133 | 2580 |
| HUALLAGA | 25598 | 11 | 303 |
| HUAMALIES | 76662 | 24 | 3436 |
| HUAMANGA | 285334 | 92 | 2760 |
| HUANCA SANCOS | 10385 | 4 | 3422 |
| HUANCABAMBA | 127330 | 30 | 1933 |
| HUANCANE | 66441 | 24 | 3848 |
| HUANCAVELICA | 161436 | 38 | 3679 |
| HUANCAYO | 511035 | 143 | 3245 |
| HUANTA | 111716 | 29 | 2642 |
| HUANUCO | 313232 | 87 | 1898 |
| HUARAL | 195491 | 53 | 186 |
| HUARAZ | 169477 | 68 | 3038 |
| HUARI | 62965 | 23 | 3110 |
| HUARMEY | 31178 | 8 | 12 |
| HUAROCHIRI | 83461 | 15 | 2380 |
| HUAURA | 223468 | 46 | 37 |
| HUAYLAS | 56775 | 25 | 2278 |
| HUAYTARA | 23025 | 4 | 2712 |
| ICA | 370775 | 47 | 409 |
| ILO | 72007 | 52 | 13 |
| ISLAY | 52353 | 13 | 52 |
| JAEN | 199773 | 38 | 753 |
| JAUJA | 82483 | 22 | 3389 |
| JORGE BASADRE | 8761 | 3 | 596 |
| JULCAN | 30588 | 28 | 3412 |
| JUNIN | 24695 | 10 | 4113 |
| LA CONVENCION | 180859 | 6 | 1063 |
| LA MAR | 89534 | 21 | 2647 |
| LA UNION | 14246 | 3 | 2675 |
| LAMAS | 86402 | 17 | 791 |
| LAMBAYEQUE | 302159 | 31 | 20 |
| LAMPA | 52500 | 9 | 3873 |
| LAURICOCHA | 38880 | 21 | 3485 |
| LEONCIO PRADO | 135628 | 27 | 648 |
| LIMA | 9174855 | 3508 | 161 |
| LORETO | 72624 | 1 | 98 |
| LUCANAS | 69066 | 5 | 3221 |
| LUYA | 51940 | 16 | 2307 |
| MANU | 25046 | 1 | 527 |
| MARAÑON | 33122 | 7 | 2893 |
| MARISCAL CACERES | 50727 | 3 | 282 |
| MARISCAL LUZURIAGA | 23773 | 33 | 3371 |
| MARISCAL NIETO | 83141 | 10 | 1417 |
| MARISCAL RAMON CASTILLA | 75013 | 2 | 74 |
| MAYNAS | 560767 | 7 | 91 |
| MELGAR | 77697 | 12 | 3918 |
| MOHO | 25920 | 26 | 26 |
| MORROPON | 156573 | 41 | 92 |
| MOYOBAMBA | 153846 | 41 | 878 |
| NASCA | 59286 | 11 | 11 |
| NAZCA | 59286 | 11 | 585 |
| OCROS | 11036 | 6 | 3311 |
| OTUZCO | 92388 | 44 | 2660 |
| OXAPAMPA | 95942 | 5 | 1806 |
| OYON | 23208 | 12 | 3619 |
| PACASMAYO | 106019 | 94 | 48 |
| PACHITEA | 77320 | 25 | 2772 |
| PADRE ABAD | 60774 | 7 | 300 |
| PAITA | 133161 | 77 | 36 |
| PALLASCA | 30698 | 15 | 3231 |
| PALPA | 12219 | 10 | 351 |
| PARINACOCHAS | 33567 | 6 | 3178 |
| PARURO | 30771 | 16 | 3068 |
| PASCO | 157882 | 29 | 4342 |
| PATAZ | 90008 | 21 | 3290 |
| PAUCAR DEL SARA SARA | 11087 | 5 | 2518 |
| PAUCARTAMBO | 51605 | 8 | 3005 |
| PICOTA | 45881 | 21 | 223 |
| PISCO | 137992 | 35 | 15 |
| PIURA | 1873025 | 53 | 36 |
| POMABAMBA | 29560 | 32 | 3057 |
| PUERTO INCA | 31491 | 3 | 210 |
| PUNO | 252348 | 39 | 3848 |
| PUTUMAYO | 12159 | .27 | 106 |
| QUISPICANCHI | 90184 | 12 | 3158 |
| RECUAY | 19339 | 8 | 3398 |
| REQUENA | 75191 | 2 | 2 |
| RIOJA | 132735 | 52 | 841 |
| RODRIGUEZ DE MENDOZA | 31489 | 13 | 1584 |
| SAN ANTONIO DE PUTINA | 71385 | 22 | 3861 |
| SAN IGNACIO | 149452 | 30 | 1303 |
| SAN MARCOS | 54628 | 40 | 2252 |
| SAN MARTIN | 192696 | 34 | 280 |
| SAN MIGUEL | 55458 | 22 | 2659 |
| SAN PABLO | 23218 | 35 | 2381 |
| SAN ROMAN | 301632 | 132 | 3832 |
| SANCHEZ CARRION | 157912 | 64 | 3185 |
| SANDIA | 73013 | 6 | 2249 |
| SANTA | 444526 | 111 | 13 |
| SANTA CRUZ | 45181 | 32 | 2034 |
| SANTIAGO DE CHUCO | 62176 | 23 | 3127 |
| SATIPO | 284848 | 15 | 628 |
| SECHURA | 77633 | 12 | 15 |
| SIHUAS | 30393 | 21 | 2784 |
| SUCRE | 11992 | 7 | 3508 |
| SULLANA | 321887 | 59 | 64 |
| TACNA | 325652 | 40 | 585 |
| TAHUAMANU | 14483 | 1 | 245 |
| TALARA | 133547 | 48 | 5 |
| TAMBOPATA | 104158 | 3 | 205 |
| TARATA | 7697 | 3 | 3084 |
| TARMA | 107621 | 39 | 3059 |
| TAYACAJA | 108451 | 32 | 3251 |
| TOCACHE | 72447 | 12 | 502 |
| TRUJILLO | 985275 | 558 | 34 |
| TUMBES | 164818 | 93 | 23 |
| UCAYALI | 75217 | 3 | 134 |
| URUBAMBA | 65410 | 45 | 2869 |
| UTCUBAMBA | 118956 | 31 | 446 |
| VICTOR FAJARDO | 23680 | 10 | 3102 |
| VILCAS HUAMAN | 23362 | 20 | 3482 |
| VIRU | 122623 | 38 | 76 |
| YAROWILCA | 32864 | 45 | 3254 |
| YAULI | 40588 | 11 | 3725 |
| YAUYOS | 27415 | 4 | 2900 |
| YUNGAY | 59207 | 43 | 2463 |
| YUNGUYO | 47476 | 164 | 3839 |
| ZARUMILLA | 55196 | 74 | 14 |

**Supplementary table 2**. Poisson regression analysis of COVID-19 cases by density population and 1000 x cases/inhabitants

|  | Altitude (m) | | | |  |
| --- | --- | --- | --- | --- | --- |
|  | <1500 | 1500 - 2499 | 2500-3499 | ≥3500m |  |
|  |  | IRR (95% CI) | IRR (95% CI) | IRR (95% CI) | p for trend |
| Total population |  |  |  |  |  |
| COVID-19 cases/density population | 1.00 (ref.) | 0.28 (0.27;0.29) | 0.10 (0.08;0.09)) | 0.14 (0.14;0.15) | <0.001 |
| 1000 x COVID-19 cases/inhabitants | 1.00 (ref.) | 0.60 (0.54;0.64) | 0.39 (0.37;0.42) | 0.40 (0.36;0.43) | <0.001 |
| Men |  |  |  |  |  |
| 1000 x COVID-19 cases/inhabitants | 1.00 (ref.) | 0.50 (0.31;0.81) | 0.40 (0.29;0.54) | 0.45 (0.29;0.71) | <0.001 |
| Women |  |  |  |  |  |
| 1000 x COVID-19 cases/inhabitants | 1.00 (ref.) | 0.62 (0.33;1.15) | 0.40 (0.26;0.63) | 0.43 (0.22;0.85) | <0.001 |

**Supplementary table 3**. Poisson regression analysis of COVID-19 mortality by density population and 1000 x cases/inhabitants

|  | Altitude (m) | | | |  |
| --- | --- | --- | --- | --- | --- |
|  | <1500 | 1500 - 2499 | 2500-3499 | ≥3500m |  |
|  |  | IRR (95% CI) | IRR (95% CI) | IRR (95% CI) | p for trend |
| Total population |  |  |  |  |  |
| COVID-19 cases/density population | 1.00 (ref.) | 0.38 (0.31;0.48) | 0.14 (0.12;0.17) | 0.24 (0.19;0.31) | <0.001 |
| 1000 x COVID-19 cases/inhabitants | 1.00 (ref.) | 0.56 (0.33;0.95) | 0.40 (0.28;0.57) | 0.46 (0.27;0.78) | <0.001 |
| Men |  |  |  |  |  |
| 1000 x COVID-19 cases/inhabitants | 1.00 (ref.) | 0.50 (0.31;0.81) | 0.40 (0.29;0.54) | 0.45 (0.29;0.71) | <0.001 |
| Women |  |  |  |  |  |
| 1000 x COVID-19 cases/inhabitants | 1.00 (ref.) | 0.62 (0.33;1.15) | 0.40 (0.26;0.63) | 0.43 (0.22;0.85) | <0.001 |

A B

C D

**Supplementary Figure 1.** Pearson residuals and deviance residuals vs fitted value: predicted mean COVID-19 cases/density population (A and B) or 1000xCOVID-19 cases/inhabitants (C and D)

C D

A B

**Supplementary Figure 2.** Pearson residuals and deviance residuals vs fitted value: predicted mean COVID-19 mortality/density population (A and B) or 1000xCOVID-19 mortality/inhabitants (C and D)

**Supplementary Figure 3.** Association between COVID-19 cases in Peru in relation to altitude (m): (A) total population, (B) men, and (C) women.

A

B

C

C

B

A

**Supplementary Figure 4.** Negative binomial regression analyses of COVID-19 cases (1000 x COVID-19 cases/inhabitants) in Peru in relation to altitude (m): total population (A), men (B), and women (C).

*IRR, Incidence-rate ratio; 95% CI, 95% Confidence Interval*

A

B

C

**Supplementary Figure 5.** Association between COVID-19 mortality 1000 x COVID-19 mortality cases/inhabitants) in Peru in relation to altitude (m): total population, (A) men (B), and women (C).

A

B

C

**Supplementary Figure 6.** Poisson regression analyses of COVID-19 mortality (1000 x COVID-19 cases mortality/inhabitants) in Peru in relation to altitude (m): total population (A), men (B) and women (C).

*IRR, Incidence-rate ratio; 95% CI, 95% Confidence Interval*
